# LIFE SATISFACTION AMONG BLACK AND WHITE AMERICANS: FINDINGS FROM THE 2020 HEALTH AND RETIREMENT STUDY

**DOI:** 10.1093/geroni/igae098.3538

**Published:** 2024-12-31

**Authors:** Chesney Ward

**Affiliations:** Utah State University, Logan, Utah, United States

## Abstract

The U.S. population is living longer, yet data are limited regarding life satisfaction among older adults. Understanding this construct for older adults is essential given the greater longevity and notable risks of reduced satisfaction as one ages. The purpose of this study was to examine racial differences between middle-aged and older White and Black Americans on life satisfaction and explore the correlation of religious beliefs and perceived control over one’s life and health with self-reported life satisfaction. The study presents results utilizing data from the 2020 Health and Retirement Study, a nationally representative sample. The sample (N=1163) was a comparative analysis of Black and White respondents. Findings indicated factors that contribute to overall life satisfaction, differences in race, and gender differences. Black respondents when compared to White respondents reported lower overall life satisfaction but higher religious beliefs. The current study helps to fill this gap by examining the relationship between psychosocial and demographic factors and life satisfaction among the sample. By drawing on the literature and the study data, implications on the impact of religious belief on overall life satisfaction are discussed. This study proposes ways in which researchers, practitioners, and policy advocates can improve life satisfaction for Black and African American populations in the United States by employing a religious aspect.

